# Experimental Evaluation of Carbon Reinforced TRC with Cement Suspension Matrix at Elevated Temperature

**DOI:** 10.3390/polym14112174

**Published:** 2022-05-27

**Authors:** Richard Fürst, Petr Hejtmánek, Tomáš Vlach, Jakub Řepka, Vladimír Mózer, Petr Hájek

**Affiliations:** 1 Faculty of Civil Engineering, Czech Technical University in Prague, 166 29 Prague 6, Czech Republic; petr.hejtmanek@cvut.cz (P.H.); tomas.vlach@cvut.cz (T.V.); jakub.repka@cvut.cz (J.Ř.); vladimir.mozer@cvut.cz (V.M.); petr.hajek@cvut.cz (P.H.); 2 Federal Institute for Materials Research and Testing (BAM), Division 7.3-Fire Engineering, Unter den Eichen 87, 12205 Berlin, Germany; 3 Fire Laboratory, University Centre for Energy Efficient Buildings of Czech Technical University in Prague, Trinecka 1024, 273 43 Bustehrad, Czech Republic; 4 Laboratory of Composite Structures, University Centre for Energy Efficient Buildings of Czech Technical University in Prague, Trinecka 1024, 273 43 Bustehrad, Czech Republic

**Keywords:** cement matrix, cement suspension, carbon fibers, textile reinforced concrete, high-performance concrete, elevated temperature, fire safety, fire resistance

## Abstract

Textile-reinforced concrete (TRC) is a new composite material comprising high-performance concrete and textile reinforcement from textile yarns with a matrix, usually consisting of epoxy resins (ER). The most significant advantage of ER is the homogenization of all filaments in the yarn and full utilization of its tensile potential. Nevertheless, ER matrix is a critical part of TRC design from the perspective of the fire resistance due to its relatively low resistance at temperatures of approximately 120 °C. This work expands the previously performed mechanical tests at normal temperatures with cement suspension (CS) as a non-combustible material for the yarn matrix. Here, the mechanical properties of CS matrix at elevated temperatures were verified. It was found that the addition of polypropylene fibers into HPC negatively affected the mechanical results of CS matrix specimens. Simultaneously, thermal insulation effect of the covering layers with different thicknesses did not significantly influence the residual bending strength of specimens with CS matrix and achieved similar results as reference specimens. Furthermore, all specimens with ER matrix progressively collapsed. Finally, CS as a textile reinforcement of yarn matrix appears to be a suitable solution for increasing the temperature resistance of TRC structures and for substituting synthetic resins.

## 1. Introduction

Textile-reinforced concrete (TRC) is currently used mainly for non-load bearing structures, design elements, and for repairing or improving existing constructions [1,2,3,4,5]. However, the requirements for sustainability and resilience of structures are significantly higher than in previous years, especially regarding structure design efficiency, better material properties or usage of materials with lower carbon footprint [6,7]. The combination of high-performance concrete (HPC), textile reinforcement, and its synthetic matrix allows to implement subtler structures with mechanical properties comparable to traditional reinforced concrete structures. Moreover, when higher quality materials with better mechanical properties are used, it is possible to reduce the total quantity of used materials and decrease the final construction’s carbon footprint [8,9,10]. In addition, TRC achieves excellent mechanical properties at ambient temperature (20 °C) and has a favorable impact on the environment [8,9,10]. 

Commonly used textile-reinforced concrete consists of HPC, tensile reinforcement consisting of carbon-fiber-reinforced polymer (CFRP) bars, and textile reinforcement as shear reinforcement. Based on the combination of these materials with better mechanical properties than materials used for traditional reinforced structures, it is possible to reduce significant quantity of used materials and thus create structures with equal load-bearing capacities. TRC has not commonly been used for load-bearing structures so far, however, due to its excellent mechanical properties, the use of this composite material is expected also for load-bearing structures in the future. For this reason, a detailed assessment of TRC from a fire-resistance point of view is required. Several studies have already investigated the behavior of TRC at elevated temperatures. However, the exposure method was primarily based on isothermal temperature exposure [11,12,13]. Therefore, for the subsequent investigation, it is necessary to focus on temperature exposure conditions representing real fire situations. The exposure method fulfilling this condition reflects the standard temperature curve, according to EN 1363-1.

The main component of TRC is HPC exhibiting excellent mechanical properties like compressive strength at ambient and elevated temperatures. Nevertheless, using HPC is often associated with a lower water content due to the use of plasticizers. Moreover, it implies low porosity of the hardened concrete, contributing to the higher probability of spalling from a fire-resistance point of view. 

**Figure 1:**
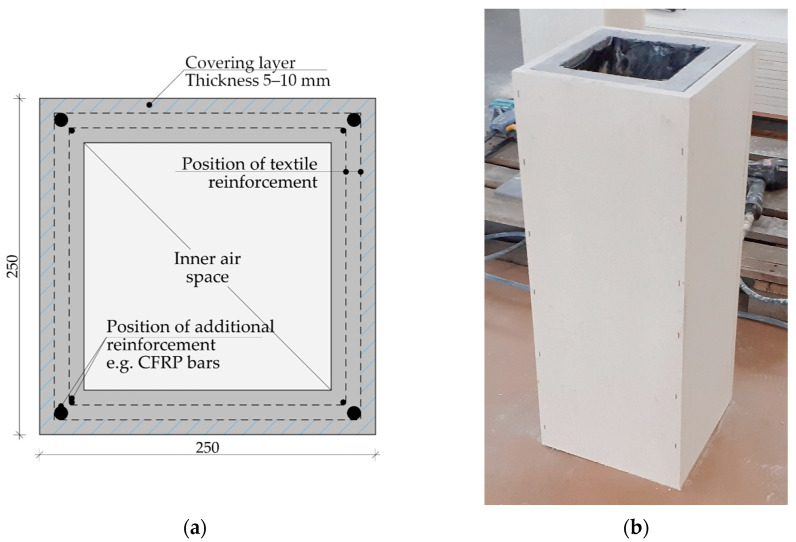
Scheme of the TRC beam structure (column) with hollow section 250 mm × 250 mm: (**a**) theoretical scheme of the column with additional CFRP bars reinforcement; (**b**) developed pattern of the TRC column with two layers of textile reinforcement and CFRP tensions bars covered by fire protection board system.

Textile reinforcement constitutes the second part of TRC and the most commonly used materials are carbon, glass or aramid [14,15]. Due to the excellent resistance to atmospheric corrosion of textile reinforcement, it is possible to design the covering layers considering only mechanical interaction between materials. Consequently, the final covering layer thickness is around 5 to 10 mm [16] (Figure 1), making the final size of elements smaller, thus reducing the consumption of silicate material for matrix (with associated environmental savings).

The usage of thinner covering layers usually attaining approximately 10 mm, makes the spalling of concrete layers more important. The spalling of the surface covering layers may fundamentally affect the load-bearing capacity of the affected structural element. To avoid the spalling risk, surface reinforcing mesh or the addition of appropriate fibers with a low melting temperature may be added into the concrete mixture, as per EN 1992-1-2. However, it is often not possible to use surface reinforcement due to the thinner cover layer. Therefore, polypropylene (PP) fibers are most commonly used in the HPC mixture to reduce the risk of concrete layer spalling [17,18]. Additionally, the subtle character of TRC structures and corresponding thinner cover layers do not provide sufficient thermal insulation. Therefore, the temperature rises significantly faster in contrast to the traditional reinforced concrete structures, where the covering layers are designed according to EN 1992-1-2, i.e., from 20 to 50 mm. Consequently, the reinforcing carbon fibers mesh degrades faster due to the higher temperature gradient and lower thermal mass of the TRC structure. 

In general, carbon fibers have the best mechanical properties among other materials used as textile reinforcement. They also have the highest melting point compared to other materials, usually exceeding the temperatures attained during a fire (Table 1). The real fire conditions are simulated based on EN 1363-1 according to the ISO 834 standard temperature curve. The reached temperatures correspond to the duration of the fire and typically exceed 1000 °C. For this reason, the use of carbon reinforcement seems to be the most suitable solution at ambient and elevated temperatures because the risk of reinforcement deterioration is minor. Moreover, carbon-based materials are characterized as materials with the lowest energy requirements for its production. In contrast to carbon materials, basalt fibers generally have the lowest carbon footprint while maintaining a higher melting temperature than glass materials [8], as compared in Table 1. For this reason, basalt appears to be a possible alternative for substituting carbon fibers.

**Figure 2:**
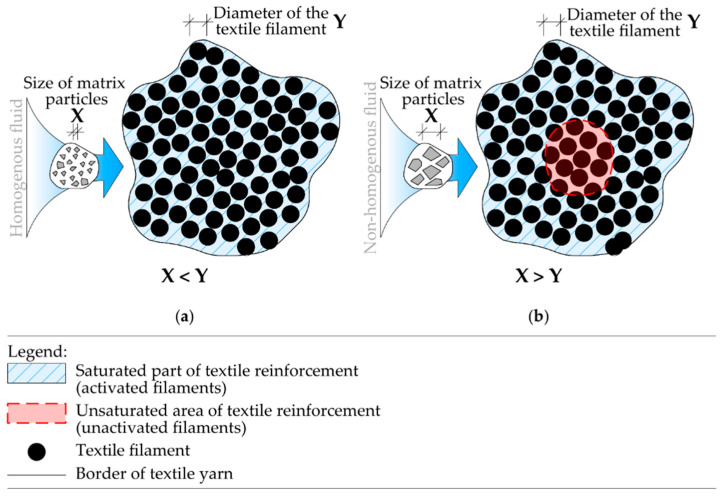
Scheme of tensile reinforcement saturation: (**a**) textile reinforcement penetrated by material with a homogenous structure such as epoxy resin—full activation of yarns is achieved; (**b**) textile reinforcement penetrated by suspension with particles larger than the filaments of the yarn—central part of yarn is not activated.

The mechanical properties of textile reinforcement can be improved by a matrix, most commonly from synthetic resins. The matrix ensures redistribution of tensile stress into the whole textile yarn and allows full utilization of the textile reinforcement, resulting in better mechanical properties. Best results were observed with simple epoxy resin curing process and homogenous consistency, enabling full penetration of the tensile reinforcement [22,23]. On the other hand, increased brittleness was observed for textile reinforcement penetrated with epoxy resins. This implies brittle behavior when the tensile strength of reinforcement is exceeded. Therefore, structures reinforced by textile reinforcement with synthetic matrix attain lower ductility than traditional construction elements reinforced by steel reinforcement. An organic textile matrix can be substituted by inorganic materials like cement, cement suspensions, and mortars [24,25,26,27]. However, these materials do not achieve as excellent results as synthetic resins, primarily due to the non-homogeneity of penetration fluid. The resulting utilization of reinforcement is hence reduced (Figure 2).

The temperature resistance of synthetic resins is represented by a temperature interval called “glass transition temperature interval”. When this temperature interval is exceeded, the modulus of elasticity rapidly decreases, and deformations occur [28]. For commonly used resins, the glass transition temperature equals approximately to 80–120 °C. Due to the low temperature resistance, the synthetic resin matrix proves to be the weakest point of the TRC design from the perspective of fire resistance. Another possibility is to substitute it by synthetic resins with significantly higher temperature resistance exceeding, approximately, 240 °C [29]. However, the curing process of these resins is often significantly complicated and usually requires elevated pressure and temperatures. For this reason, the use of these products with higher temperature resistance is often inappropriate due to technological processes in the construction of structures in civil engineering. Furthermore, these organic synthetic resins can also theoretically contribute to pressure increase within the structure due to the resin thermal decomposition products and subsequently negatively affect the spalling risk. Simultaneously, these decomposition products may also contribute to the development of fire [30,31]. 

In general, the solution for preventing negative impact of synthetic resins during fire is to apply additional fire protection layers, i.e., boards or plasters. These protection systems can avoid the undesirable increase of temperature in the construction and thus prevent a weakening or collapse of the whole structure. The subtle character and environmental advantage will be infringed when an additional fire protection layer is applied. A suitable solution can be when materials with better temperature resistance are used instead of the additional fire protection systems, thus maintaining better carbon footprint, economic efficiency, and subtle character of the TRC structures.

A suitable solution can also be the use of non-flammable inorganic materials instead of synthetic resins. Previous research verified that cement suspension with CEM 52.5 R achieved an approximately 30% improvement in comparison with reference specimens with epoxy resin [32]. Although cement suspensions do not achieve such results at ambient temperature as synthetic resins, they significantly exceed their temperature resistance at elevated temperatures. For this reason, this work aims to demonstrate a comparison of the behavior of organic (epoxy resin) and inorganic (cement suspension) textile reinforcement matrices at elevated temperatures, according to the ISO temperature curve. The test specimens in this work were designed with carbon reinforcement, which achieved satisfactory temperature resistance and allowed to separate the influence of the textile reinforcement matrix at elevated temperatures for both matrix variants. Test specimens were also designed with and without PP fibers in the HPC mixture. A four-point bending test verified the resulting change in mechanical properties, including comparison with reference specimens that have not been exposed to fire. 

Given the above, the main objective of this work is the determination of the interaction effect between the textile reinforcement and HPC in comparison to samples achieving high temperatures during the fire tests. The proposed hypothesis that cement-based suspension has sufficient performance to be a possible replacement for epoxy resin, will be tested trough the proposed experimental evaluation and result analysis. This will provide the basis for a more effective design of load-bearing structures made from TRC without additional fire protection and describe the behavior of TRC with inorganic textile reinforcement matrix at elevated temperatures.

## 2. Materials and Methods

### 2.1. Test Specimens 

The geometry of test specimens was chosen to reflect the application in subtle-walled structures, such as load-bearing wall panels, columns with a hollow cross-section or facade elements. Therefore, the dimension selected are 100 mm × 360 mm with a thickness of 30 mm. The test specimen series were designed with a different covering layer thicknesses, textile reinforcement matrix material, and the addition of PP fibers in the HPC mixture. Each series contained five identical samples that were exposed to elevated temperatures. At the same time, reference specimens were created for each test series. For the description and labelling of the test series, see Table 2. 

**Figure 3:**
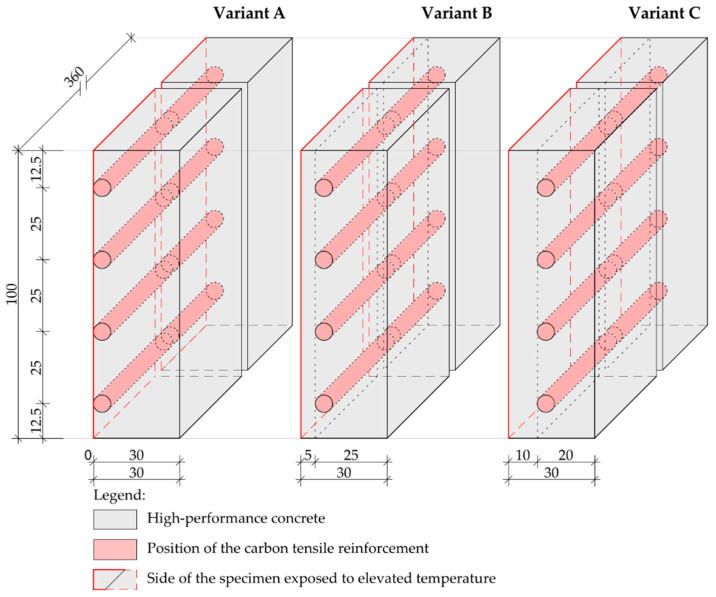
Scheme of the test plate specimens and position of the tensile reinforcement Legend: Variant A—plate specimens with 0 mm covering layer used for direct exposure of textile reinforcement to fire Variant B—5 mm covering layer as minimum possible covering layer regarding the interaction between the materials Variant C—standard 10 mm covering layer of TRC structures.

During the development of specimens, the tensile reinforcement layer was placed in different positions, depending on the specific specimen series (Figure 3). The thickness of the covering layer varied from 0 mm to 10 mm (Table 2). The corresponding covering layer position was defined by fixing the saturated reinforcing yarns in the mould walls. At the same time, accompanying beam and cube specimens for the determination of mechanical properties of HPC according to EN 12395-3 and EN 12390-5 were created. The dimensions of specimens were 40 mm × 40 mm ×160 mm and 100 mm × 100 mm × 100 mm.

Two different variants of textile reinforcement were used. The first variant contained a prefabricated carbon grid saturated by an epoxy resin matrix. This commercial product is available as Q85/85-CCE-21 from the Solidian company. The second variant of specimens used carbon rovings Tenax^®^ E HTS 40. The second variant of reinforcement was subsequently impregnated with a cement suspension. The suspension was prepared from cement CEM 52.5 R according to [32] with water with a ratio of 1:2. Several rollers mechanically pressed and pulped the carbon yarn, which enabled deeper penetration of the cement suspension. Related mechanical properties of the textile reinforcement were drawn from the technical sheets of products and described in Table 3. 

The HPC mixture developed at CTU in Prague was chosen for specimens’ development. Based on the corresponding type of test specimens described in Table 2, the related variant of HPC mixtures provided in Table 4 was used. The mechanical properties of the cured concrete attained approximately the compressive strength of 110 MPa, tensile strength of 11 MPa with the modulus of elasticity equal to 49.5 GPa. 

After the tensile reinforcement was placed in the correct height position, wire thermocouples were attached to the surface of the reinforcement layer. To prevent the shifting of the thermocouple’s measuring end, all thermocouples were mounted on a support construction independent of the test specimen’s mould. After 24 h, specimens were removed from the mould and subsequently conditioned for 28 days according to EN 12390-2 in the lime-water bath.

### 2.2. Fire Tests Instrumentation 

All fire tests were carried out in a mobile fire furnace called miniFUR developed at CTU in Prague. The supporting structure of the test furnace is made of steel profiles covered by cement-fiber panels. The dimensions of the furnace combustion space are 800 mm × 800 mm × 1200 mm. Natural furnace ventilation is ensured through two inlet openings at the furnace bottom and two exhaust openings. The size of all vents is 100 mm × 300 mm. Temperature distribution within the fire furnace is accomplished by a sand burner positioned in the center. The fuel used is propane 2.5 and it is possible to apply temperature curves in the heated space corresponding to ISO 834 according to EN 1363-1. 

**Figure 4:**
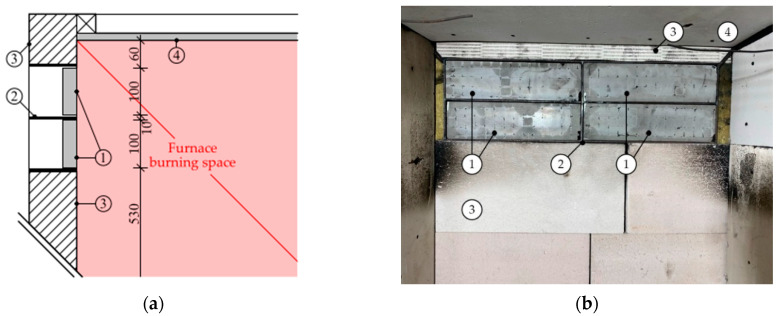
Scheme of the position of the test specimens: (**a**) side view on the specimen placements; (**b**) view from inside the furnace of the placed test specimens Legend: 1—exposed side of the test specimen; 2—steel construction holding test specimens; 3—furnace front wall from aerated concrete blocks thickness 100 mm; 4—ceiling of the furnace.

There were 8 specimens tested at once: In both short ends of the furnace, aerated concrete block walls were mounted. In the wall, in the height of 530 mm above the furnace floor, steel frames were placed to accommodate and hold 4 specimens in position. An expansion space between the test specimen and the holding steel construction was left to prevent mechanical stress due to the thermal expansion of specimens. The surroundings around the steel structure with test specimens were protected by thermal insulation from mineral wool and aerated concrete blocks (Figure 4).

During the fire tests, the gas temperature was measured using eight sheathed thermocouples Ø 3.0 mm, type K. The thermocouples were placed at 300 mm and 600 mm from the furnace floor. Based on the measured data, the temperature course in the burning space was compared and controlled according to the temperature curve limits specified in EN 1363-1 for ISO 834. The temperature of the test specimens was monitored by wire thermocouples in the textile reinforcement layer. Data acquisition for all measured temperatures was provided through a measuring control panel, module EDAM-5019.

### 2.3. Mechanical Testing

The mechanical properties of the hardened concrete HPC mixture were determined according to EN 12395-3 and EN 12395-5. Tensile strength of the HPC was determined by the three-point bending test. Resulting tensile strength was calculated according to Equation (1).

f_cf_ = (3·F_t_·l)/(2·d_1_·d_2_^2^) (1)

where: f_cf_—bending tensile strength (MPa); F_t_—measured force value during the mechanical test; l—distance between supports (100 mm); d_1_ and d_2_ width of samples (mm). The resulting value was rounded to the nearest 0.1 MPa.

The compressive strength was determined as an average of the results from tested HPC cubes 100 mm × 100 mm × 100 mm and fractions of the beam samples from three-point bending tests according to equation 2.

f_c_ = F_t_/A_c_ = F_t_/(d_1_·d_2_) (2)

where: f_c_—compression strength (MPa), F_t_—maximum load value during the compression test (MPa); d_1_ and d_2_—the sample cross-sectional area (mm). The resulted value was rounded to the nearest 0.5 MPa.

**Figure 5:**
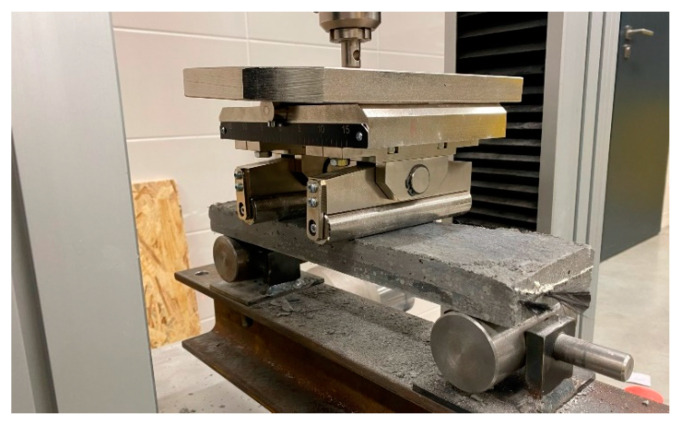
Test specimen place in the mechanical press for four-point bending test.

The bending strength of the reference plate specimens and residual bending strength of specimens exposed to elevated temperature was determined by a four-point bending test (Figure 5). The maximal applied force was measured during the mechanical test before the rupture, or when massive deformation of samples has occurred. The force applied during the mechanical test was transferred through a steel element with three degrees of freedom to eliminate geometric imperfections in the test body and to ensure uniform loading. The distance between the steel cylinders of mechanical press was 100 mm. The velocity of applied force was set at 2 mm·min^−1^. The entire mechanical test was carried out according to EN 12390-5. 

The four-point bending mechanical tests were performed in the appropriate mechanical press Galbadini Quasar 10 kN. For test specimens with an epoxy resin matrix, Galbadini Quasar 100 kN was used. Based on the technical data sheets of measurement devices, the accuracy of measurements for both presses was determined as class 1 accuracy with a deviation ±1% from the maximal load-cell value (according to EN ISO 7500/1). 

### 2.4. Statistical Evaluation Data Analysis

For measured gas temperature and temperature of the specimens exposed to fire, the expanded measurement uncertainty was determined with uncertainty coefficient k_u_ = 2.0 for the interval containing 95% of values. All statistical evaluation was performed in MS Excel 365 and GraphPad Prism 8.0. 

## 3. Results and Discussion

### 3.1. Testing at Ambient Temperature 

PP fibers have been added to the HPC mixture to reduce the risk of spalling of concrete layers according to EN 1992-1-2. Due to the PP fibers added in the HPC mixture, the final compressive strength was reduced by 9% and tensile strength by 2%. The resulting compressive strength of used HPC with/without PP fibers measured was 109.6/119.4 MPa, while for tensile strength of HPC with/without PP fibers it was 12.5/13.0 MPa. 

**Figure 6:**
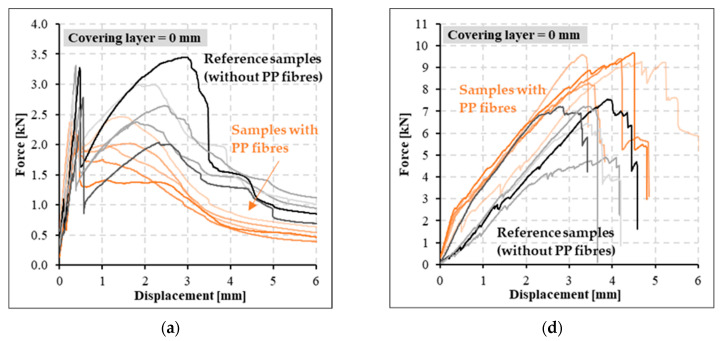

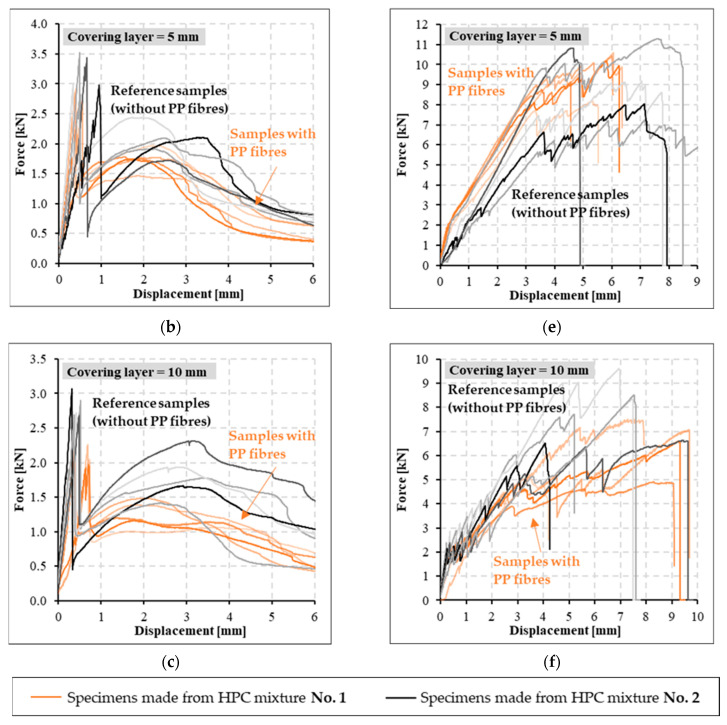
Bending strength of reference specimens with cement suspension matrix (**a**–**c**) and epoxy resin matrix (**d**–**f**) for different thickness of the covering layer.

The bending strength of all reference specimens was determined in four-point bending tests. Based on these mechanical tests, the negative influence of PP fibers in the concrete mixture on specimens, especially with cement suspension matrix (CPP series), was observed (Figure 6a–c). The maximum measured strength peak of specimens with cement matrix was shifted approximately 1 mm closer to the initial concrete crack at the tensile surface of the specimens. Concurrently, the maximum measured value of bending strength was approximately 24% less than for specimens without PP fibers (C series). In contrast, in test specimens with an epoxy resin matrix (SPP series), a significant effect of PP on the attained bending strength was not observed, as was the case for samples with cement suspension matrix (Figure 6d–f). Simultaneously, a different pattern of specimen failure can be observed. In specimens with a cement suspension matrix, the reinforcing carbon yarns were pulled out, and progressive collapse did not occur. On the contrary, the specimens with epoxy resin matrix were broken after reaching the tensile strength of carbon reinforcement.

### 3.2. Testing at Elevated Temperatures 

**Figure 7:**
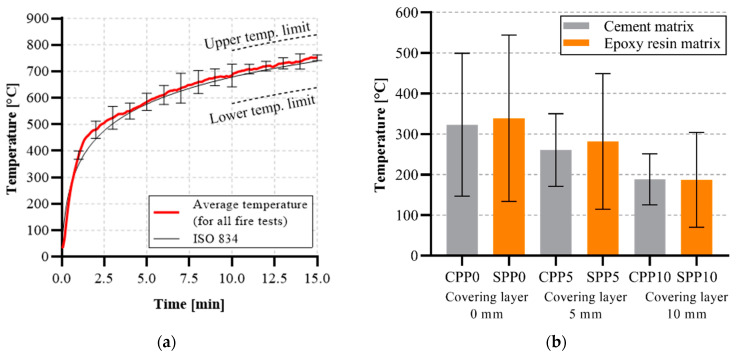
Measured temperature from the test furnace and tested specimens: (**a**) average temperature curve in the test furnace; (**b**) measured average temperatures of the specimens on the inner surface of the textile reinforcement for different covering layers and concrete mixture variants.

In the second phase of experimental work, fire tests were performed. Each series of test specimens, excluding reference specimens, was exposed to the ISO 834 standard temperature curve according to EN 1363-1 (Figure 7a). A higher difference between the actual measured gas temperature and the ISO temperature curve was observed from the 5th to 8th minute. Nevertheless, the temperatures did not exceed the ISO temperature curve limits. For this reason, the temperature distribution in the test furnace was satisfactory for each fire test.

Temperatures measured on the unexposed side of the tensile reinforcement layer differed according to the thickness of the covering concrete layer (Figure 7b). For specimens with an epoxy resin matrix (SPP series), higher temperature was measured in the specimens of variants SPP0 and SPP5. This was caused by the complete or partial burning out of the epoxy resin material, as determined by visual analysis after the mechanical tests. In both cases (SPP-0 and SPP-5), the temperatures exceeded approximately 280 °C, implying an advanced epoxy resin decomposition phase. On the contrary, for specimens with a 10 mm cover layer (SPP-10), the temperature did not exceed 200 °C, and the epoxy resin did not burn out. The temperature difference of both samples’ series with cement and epoxy resin matrix (with covering layer of 5 and 10 mm) was approximately 20 °C. 

After fire exposure, the specimens with PP fibers (CPP/SPP series) added into the HPC mixture retained their integrity. However, for specimens without PP fibers, the destruction of all specimens occurred due to the increase of inner water vapor pressure and closed pore structure of HPC. The increased internal pressure, combined with the subtle character of specimens, has led to the loss of integrity of the entire test specimens without PP fibers before the end of the fire test. Therefore, it was impossible to determine the residual bending strength of these specimens. 

**Figure 8:**
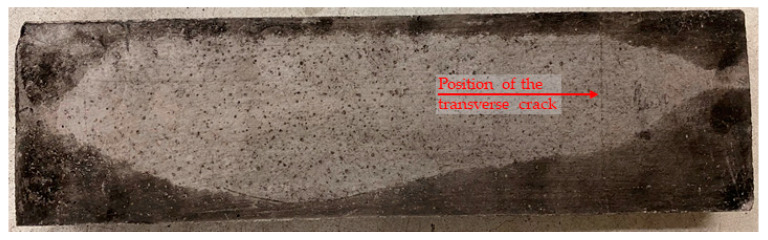
Test specimen with 10 mm covering layer and an addition of PP fibers in concrete mixture (HPC mixture No. 1)—the development of a transverse crack during a fire test.

Different behavior was observed for test specimens with the addition of PP fibers using various materials of the textile reinforcement matrix. For cement suspensions specimens, spalling of concrete layers did not occur in any test. Only transverse cracks were observed because of the fire exposure, (Figure 8).

**Figure 9:**
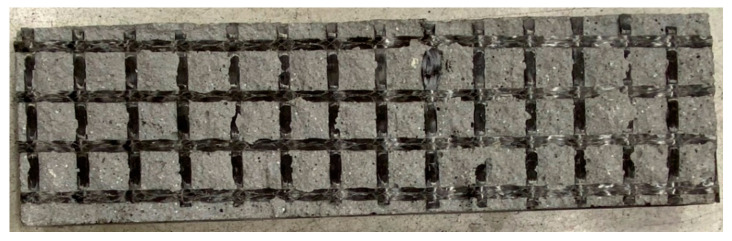
Test specimen of SPP10 series after the covering layer spalled off after the fire experiment (no direct exposure of the reinforcing layer during fire test occurred).

Similar behaviour was observed for specimens with epoxy resin matrix and no covering layer (SPP-0). The absence of spalling may be due to the reinforcing layer being placed near the fire exposed surface. This layer allowed additional reinforcement against spalling. On the other hand, no additional pressure accumulation could occur due to the thermal degradation of the epoxy resin. In contrast, for the test specimens with a covering layer of 5 and 10 mm (series SPP-5 and SPP-10), a separation of the entire area of the covering layer at the exposed surface of the textile reinforcement was observed. It occurred probably due to the influence of the epoxy resin thermal decomposition products in combination with the epoxy resin’s loss of mechanical strength and due to the phase transition. As a result, the covering layer was weakened at this point and subsequently, the covering layer spalled off. Especially specimens with a cover layer of 5 mm showed only local spalling due to the possibility of water vapor and epoxy resin thermal decomposition products migration to the outside of the test specimen. On the contrary, for specimens with a covering layer equal to 10 mm (SPP-10), a cavity was formed between the covering layer and the rest of the test specimen. In most cases, the covering layers fell off, leaving the textile reinforcement directly exposed to fire. Therefore, it was not possible to determine residual bending strength. The smooth surface character of the textile reinforcement saturated by epoxy resin is also a significant contribution to the covering layer spalling at the zone of the reinforcing mesh. Moreover, due to the phase transition of epoxy resin, the epoxy resin is losing its mechanical properties. Therefore, the adhesion between materials weakened. Only in one case of the SPP-10 test series the specimen’s integrity was maintained and it was possible to determine the residual bending strength (Figure 9).

**Figure 10:**
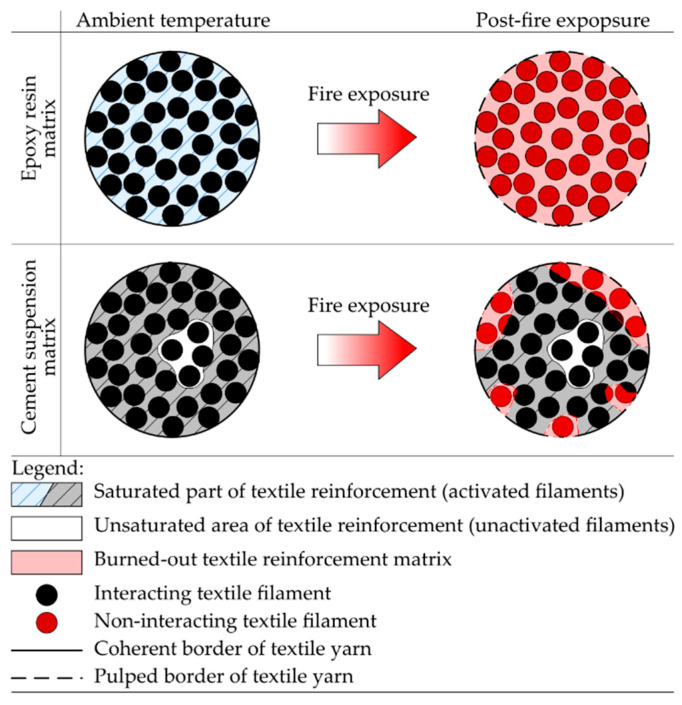
Schematic description of the fire exposure influence to a different type of textile reinforcement matrix.

Based on the four-point bending test data, the residual bending strength of specimens exposed to elevated temperatures was determined. It was observed that the textile reinforcement from epoxy resin completely burned out due to the direct fire exposure of textile reinforcement or due to exceeding the ignition temperature of epoxy resin (Figure 7b) when the covering layer did not spall. On the contrary, cement matrix was damaged on the surface only partially and the interaction between materials remain undisturbed to a certain extent (Figure 10).

**Figure 11:**
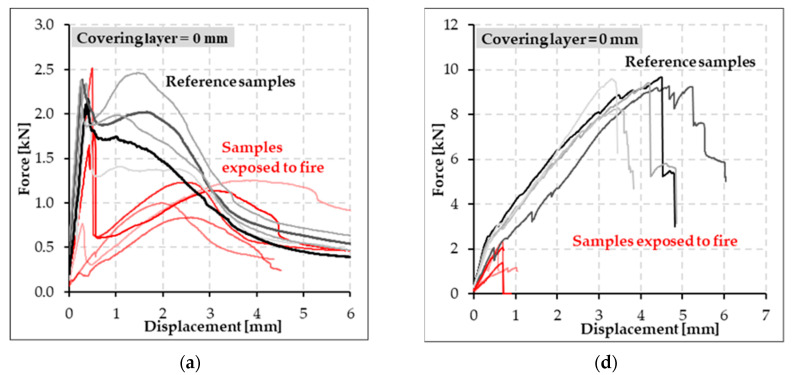

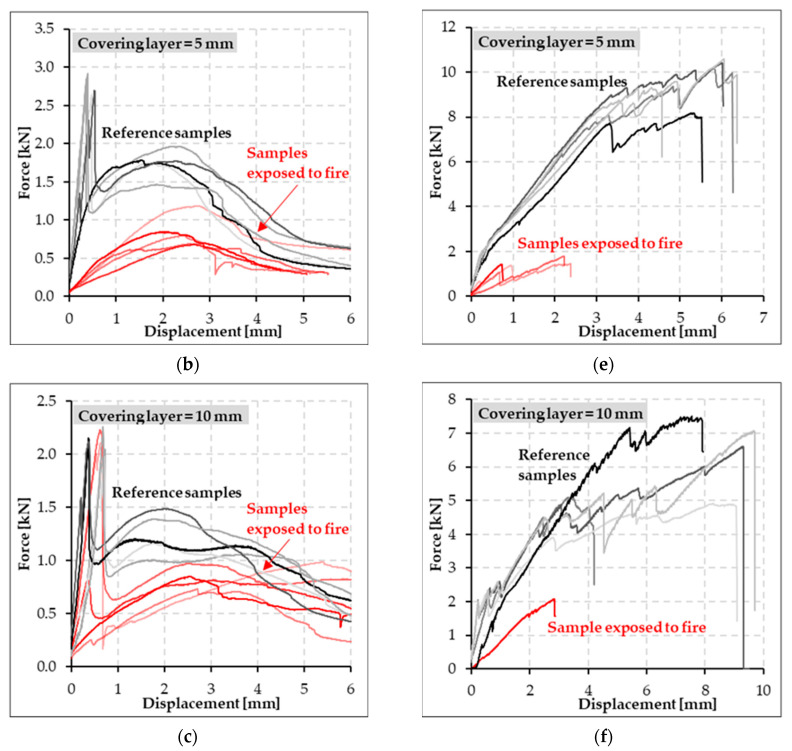
Comparison of the resulting residual strength of the test plate specimens after the fire test with reference specimens: (**a**–**c**) test plate specimens with cement suspension matrix; (**d**–**f**) test plate specimens with epoxy resin textile reinforcement matrix Note: All specimens mentioned in Figure 11 were made according to Table 4 from the HPC mixture No. 1.

Tested specimens with cement suspension matrix at elevated temperatures showed a similar trend of bending strength capacity as the reference specimens (Figure 11a–c). The results show that these specimens did not incur a significant decrease of mechanical bending strength as in the case of samples with the epoxy resin matrix. This was caused by better temperature resistance of the cement suspension matrix material. Due to temperature effects, the bending strength decreased by approximately 40%. Simultaneously, the cement suspension samples showed a significant increase in ductility. Higher ductility was caused by gradual pull out of individual filaments of the carbon yarn during the whole mechanical test. In contrast to the specimens with an epoxy resin matrix, textile reinforcement did not drop out during mechanical tests, and the progressive break did not occur.

In tested specimens with an epoxy resin matrix (SPP series), we observed a significant degradation of the epoxy resin due to the attained temperatures during the fire test (Figure 7). Therefore, a significant loss of mechanical properties of these specimens occurred (Figure 11d–f). During the fire tests, this matrix was partially burned out. Subsequently, the ability to homogenize the individual filaments in the textile yarn was infringed. In contrast to the reference specimens with epoxy resin matrix, a progressive break did not occur when the tensile strength of textile reinforcement was reached. Specimens with epoxy resin tensile reinforcement matrix exposed to elevated temperature were broken due to the pulling of textile reinforcement or breakage due to absent interaction between the textile reinforcement and HPC—the textile reinforcement disengaged from HPC.

## 4. Conclusions

Based on previous experimental work [32], where the behavior of the cement suspension matrix was analyzed at ambient temperature, the experimental investigation described in this study focused on assessing residual bending strength at elevated temperature. As part of the experimental work, plate test specimens with dimensions 100 mm × 360 mm × 30 mm with different textile reinforcement matrix materials were tested. Also, the influence of the presence of polypropylene (PP) fibers in the high-performance concrete (HPC) mixture was assessed. Based on tests at ambient and elevated temperatures, the conclusions of the study are summarized as follows:

- Bending strength of specimens with cement suspension matrix (CPP series) decreased by approximately 24% due to the addition of the PP fibers into the HPC mixture. Simultaneously, the measured peak of the bending strength and the corresponding deformation were reduced by approximately 1 mm. However, based on the fire tests, the absence of PP fibers in the HPC mixture negatively affected the mechanical properties of these specimens at elevated temperatures. All test specimens without PP fibers failed due to the massive spalling of the concrete layers, and it was not possible to determine their residual bending strength.
- Performed fire tests clearly demonstrated the positive effect of improved temperature resistance while using a non-combustible matrix of cement-based textile reinforcement. Although the test specimens did not achieve as high bending strengths as those with an epoxy resin matrix, no progressive failure occurred due to the loss of engagement among the materials after exposure to fire. Concurrently, specimens with cement suspension show significantly better performance than the samples without any matrix of textile reinforcement as described in [32]. Moreover, a similar trend of attained bending strength was observed for the reference specimens, although the residual bending strength decreased by approximately 40%.
- Tested specimens with cement suspension showed different failure modes. There was no progressive break but a gradual pulling out of the textile reinforcement. That is why these samples showed significantly higher ductility than samples with epoxy resin matrix.
- For test specimens with cement suspension matrix, spalling of the concrete layers did not occur, in contrast to the test specimens with synthetic resin matrix. Generally, epoxy resins can facilitate the spalling of the concrete layer. This is due to the originating epoxy resin thermal decomposition products during the fire test. These products can contribute to the pressure from the evaporated water and accelerate the spalling of the concrete layers. Simultaneously, upon contact with flames, these products can ignite and thus directly contribute to the development of a fire.

The study shows that using silica-based materials as a textile reinforcement matrix positively affects mechanical properties of specimens after exposure to elevated temperature. However, it is mainly a matter of finding a compromise between maximum bending potential, ductile behavior, or preservation of mechanical properties after exposure to fire. Due to the better temperature stability of silicate (non-flammable) suspension, there was no rapid decrease in residual bending strength. 

A similar trend of decreasing bending strength was described in the study [13], where the specimens with glass textile reinforcement were tested. However, in other studies evaluating TRC at elevated temperatures, it was impossible to compare the measured results effectively due to different testing methodologies [11,12]. Nevertheless, since the measured strengths were lower than reference samples at ambient temperature, it is necessary to research this topic further and verify the use of other matrix materials, such as geopolymers, or search for a different method of impregnating textile fibers. In addition, more fire tests shall be performed, in order to get results of TRC behavior that correspond to fire exposure according to realistic fire, which is in agreement with Kapsalis et al. [33].

Given the results of the experimental study presented, it may by stated that the hypothesis of suitability of cement suspension as a replacement for epoxy resin matrix has been confirmed at elevated temperatures. Nonetheless, the performance of cement-based matrix at normal temperatures is not sufficient, hence further research will be focused on noncombustible matrix materials, geopolymers in particular.

## Figures and Tables

**Table 1:** Comparison of mechanical properties and melting points of selected textile reinforcement materials.

Mechanical Attribute	Type of Textile Reinforcement			Units
	Carbon Fibers [19]	Glass Fibers [20]	Basalt Fibers [21]	
Density	~2.0	~2.5	~2.75	kg·m^−3^
Tensile strength (average)	4.3	3.5	~2.48	GPa
Modulus of elasticity (average)	240	57	76	GPa
Diameter of yarn’s filament	7–10	10–16	~12.8	µm
Melting temperature	3650	800	1100	°C

**Table 2:** Description of the test series.

Labeling of Series ^1^	Covering Layer	Tensile Reinforcement Variant
[-]	[mm]	[-]
C-0/CPP-0 S-0/SPP-0	0 0	Textile reinforcement saturated with CEM 52.5 R suspension Prefabricated textile reinforcement saturated by epoxy resin
C-5/CPP-5 S-5/CPP-5	5 5	Textile reinforcement saturated with CEM 52.5 R suspension Prefabricated textile reinforcement saturated by epoxy resin
C-10/CPP-10 S-10/SPP-10	10 10	Textile reinforcement saturated with CEM 52.5 R suspension Prefabricated textile reinforcement saturated by epoxy resin

Legend: C—cement suspension; S—Solidian prefabricated grid; ^1^ The test series marked as *x*PP-*y* indicate the addition of the PP fibers to the HPC mixture.

**Table 3:** Material data of carbon reinforcement.

Tensile Properties	Q85/85-CCE-21	E HTS 40	Units
Tensile strength	4000	4300	MPa
Modulus of elasticity	230	240	GPa

**Table 4:** HPC mixtures components.

Labeling of HPC Mixture	No. 1	No. 2
Mixture Components	Quantity	
	kg·m^−3^	
CEM I 42.5 R	650	650
Silica sand	1200	1200
Silica flour (ground quartz)	265	265
Silica fume (microsilica)	75	75
Superplasticizer	16	16
Water	180	180
Polypropylene fibers	4.0	-
Total	2390	2386

## Data Availability

The data presented in this study are available on request from the corresponding author.
